# Balance Changes in Patients With Relapsing-Remitting Multiple Sclerosis: A Pilot Study Comparing the Dynamics of the Relapse and Remitting Phases

**DOI:** 10.3389/fneur.2018.00686

**Published:** 2018-08-21

**Authors:** Oliver Findling, Heiko Rust, Özgür Yaldizli, Dionne P. H. Timmermans, Alja Scheltinga, John H. J. Allum

**Affiliations:** ^1^Department of Neurology, University of Basel Hospital, Basel, Switzerland; ^2^Department of Neurology, Cantonal Hospital Aarau, Aarau, Switzerland; ^3^Division of Brain Sciences, Imperial College London, Charing Cross Hospital, London, United Kingdom; ^4^Radboud University Nijmegen, Nijmegen, Netherlands; ^5^Division of Audiology and Neurootology, Department of ORL, University of Basel Hospital, Basel, Switzerland

**Keywords:** multiple sclerosis, balance control, trunk sway, relapse-phase multiple sclerosis, remitting-phase multiple sclerosis, EDSS scores

## Abstract

**Aims:** To compare balance changes over time during the relapse phase of relapsing-remitting multiple sclerosis (RRMS) with balance control during the remitting phase.

**Methods:** Balance control during stance and gait tasks of 24 remitting-phase patients (mean age 43.7 ± 10.5, 15 women, mean EDSS at baseline 2.45 ± 1.01) was examined every 3 months over 9 months and compared to that of nine relapsing patients (age 42.0 ± 12.7, all women, mean EDSS at relapse onset 3.11 ± 0.96) examined at relapse onset and 3 months later. Balance was also compared to that of 40 healthy controls (HCs) (age 39.7 ± 12.6, 25 women). Balance control was measured as lower-trunk sway angles with body-worn gyroscopes. Expanded Disability Status Scale scores (EDSS) were used to monitor, clinically, disease progression.

**Results:** Remitting-phase patients showed more unstable stance balance control than HCs (*p* < 0.04) with no worsening over the observation period of 9 months. Gait balance control was normal (*p* > 0.06). Relapsing patients had stance balance control significantly worse at onset compared to remitting-phase patients and HCs (*p* < 0.04). Gait tasks showed a significant decrease of gait speed and trunk sway in relapsing patients (*p* = 0.018) compatible with having increased gait instability at normal speeds. Improvement to levels of remitting patients generally took longer than 3 months. Balance and EDSS scores were correlated for remitting but not for relapse patients.

**Conclusions:** Balance in remitting RRMS patients does not change significantly over 9 months and correlated well with EDSS scores. Our results indicate that balance control is a useful measure to assess recovery after a relapse, particularly in patients with unchanged EDSS scores. Based on our results, balance could be considered as additional measurement to assess recovery after a relapse, particularly in patients with unchanged EDSS.

## Introduction

Multiple sclerosis (MS) is a chronic autoimmune inflammatory disease of the central nervous system (CNS) which can affect visual, sensory and motor systems. Balance impairment is one of the most prominent and feared symptoms of MS and can have multifactorial causes, including cognitive, visual, vestibular, sensory-motor, and coordination functions (1, 2). Neuroradiological studies found impaired structural integrity of the corpus callosum, cortico-cerebellar connectivity or reduced connectivity between cerebellar dentate nuclei and caudate nucleus in MS patients with impaired balance (3, 4). Furthermore, a delayed onset of postural muscle activity due to lesions in supraspinal structures and slowed somatosensory nervous conduction have been reported (5) as contributing factors to the balance deficits in MS patients.

Relapsing-remitting MS (RRMS) is characterized by the occurrence of clinical relapses (6). In early stages of the disease, repair on the cellular level of the CNS may contribute to recovery after a relapse, but this usually remains incomplete (7). Most RRMS patients will therefore accumulate CNS damage over time due to relapses, causing their degree of disability to increase. The most widely used instrument to monitor disease progression in MS patients is the Expanded Disability Status Scale (EDSS) (8, 9). However, despite the fact that balance is one of the most prominent symptoms of MS, there is no specific functional system score for balance among the functional system scores from which the EDSS is calculated. For the EDSS score, balance is assessed by using the Romberg test, and an evaluation of gait ataxia during normal walking and during tandem gait. These clinical tests are part of the “cerebellar functional system score” (9) which contributes to the overall EDSS score. Therefore, during balance changes, specifically during improvement following a relapse, it might be worthwhile to examine whether, in fact, quantified balance scores are correlated with EDSS scores.

It is not known to what extent balance impairments in RRMS change during the stable remitting phase after a relapse. When no relapse occurs, RRMS patients are known to have unchanged EDSS scores over several years, and can be expected to have unchanged balance control (10). Corporaal et al. showed that balance deficits in trunk sway in MS patients correlate with their EDSS scores (11). In contrast, Martin et al. reported that patients experience balance impairments while the EDSS scores are minimally altered (12). In addition, currently there are no balance assessment tools that focus on evaluating MS patients who are experiencing a relapse or relapse recovery. In daily clinical routine the assessment of a relapse is often subjective depending on the concerns of the patient. Thus, there is a need for more reliable objective methods to quantify neurological deficits in MS, not the least, because MS treatment is becoming more individualized and every new relapse should lead to a re-evaluation of the current treatment strategy (13). One method would be to use measures of balance control to assess the relapse phases of RRMS.

Balance control has been used to assess improvements in vestibular-spinal systems following a sudden peripheral vestibular loss due to vestibular neuritis (14, 15). It has also been used to measure the permanent effect of lower-leg polyneuropathy on balance during stance and gait tests (16). In the cases of vestibular neuritis, the balance control improves to the levels of healthy controls (14, 15). These improvements have a time course of <3 months with a shorter time course for stance than gait balance control improvement and provide insights into the duration of central compensation processes (14, 15). If no compensation occurred in MS relapse patients, the question arises whether the deficits in central compensation are similar to those of chronic leg proprioceptive loss patients (16). Given this background, we investigated whether the balance control and EDSS scores of RRMS patients during relapse diverge widely from those of RRMS patients during remission more than 3 months following a relapse onset. That is, how the relationship between EDSS scores and balance measures changes with respect to that of the remitting phase over the time course of a relapse and also the form of the remaining deficit at 3 months.

We performed this study to test the hypotheses that MS patients during remission with stable EDSS scores have stable balance measures over a 9-month period, and that a relapse phase leads to deterioration in balance with respect to stable scores lasting longer than 3 months. Thus, the primary objective was to compare balance during stance and gait tasks between RRMS patients during remission and those having a relapse. Remitting patients were followed four times over 9 months in order to observe whether these patients indeed had stable balance and EDSS scores. The second objective of this study was to determine whether any longitudinal balance changes in remitting patients are related to changes in EDSS scores, and whether balance changes are related to any changes in EDSS scores for patients during relapse.

## Materials and methods

### Subjects and balance tasks

For this study, nine RRMS patients at relapse onset (mean age 42.0 ± 12.7, all women, mean EDSS at relapse onset 3.11 ± 0.96) were studied. The symptoms at relapse onset are shown in Table 1. All relapse patients were treated with high-dosed steroids. Relapse patients data was compared to that of 24 RRMS patients during remission (mean age 43.7 ± 10.5, 15 women, mean EDSS at baseline 2.45 ± 1.01). Patients' balance scores were also compared to those of 40 age- and gender-matched (to remitting RRMS patients) healthy controls (mean age 39.7 ± 12.6, 25 women) selected from data reported in prior publications (17, 18). Subject details and patients' disease modifying treatment are provided in Table 2. All MS patients were diagnosed, post onset of first symptoms, according to the 2010 revised criteria of McDonald et al. (19). If any remitting RRMS patient had a relapse, confirmed by a clinical examination, during the study they were excluded from the study. Relapses were diagnosed when new or previous symptoms were reported and could be confirmed by a clinical examination. The symptoms had to exist for at least 24 h, the time interval to the previous relapse had to be at least 30 days, and the symptoms could not be explained by a change in body temperature or by infection. The first test (at onset) for relapsing patients occurred within 7 days of relapse onset. These patients were retested after 3 months in order to determine if balance had returned to levels of remitting RRMS patients within 3 months. Based on the duration of central compensation of vestibular neuritis patients (14, 15) we expected that after 3 months, balance of the MS patients would have improved to the level of remitting patients if the same central neural processes were used by the MS patients. Twenty-one RRMS patients during remission were tested in total four times, 3 months apart over 9 months. Three remitting patients were only tested three times due to scheduling problems. The 9 month follow-up for the remitting patients was chosen because we wanted to determine that the balance control of these patients was constant enough to be used as a base-line to compare with balance control of patients during relapse.

**Table 1:** Symptoms and disease modifying treatment (DMT) at relapse onset in patients with relapse.

**Patient**	**Symptoms**	**Treatment**	**Disease duration (years)**	**EDSS**
1	Paresthesia and weakness both legs and left arm	no DMT	7.4	2.5
2	Left-sided motor hemiparesis	Glatiramer acetate	3.9	3.5
3	Gait ataxia	no DMT	0.3	1.5
4	Paresthesia and weakness right leg	Fingolimod	8.9	4.0
5	Paresthesia both arm, legs and trunk	Fingolimod	20.1	3.5
6	Paresthesia and weakness left leg	no DMT	0.1	2.5
7	Right-sided sensomotor hemiparesis	no DMT	10.0	3.0
8	Paresthesia right arm, leg and trunk	Interferon beta-1a	23.9	4.5
9	Paresthesia both legs and right arm	no DMT	13.5	3.5

**Table 2:** Patient Characteristics (means and standard deviations).

**Demographics**	**During relapse**	**During remission**	**Healthy controls**
Age in years	42.8 ± 13.4 (20–65)	45.5 ± 10.8 (29–68)	39.7 ± 12.6 (27–61)
Women	9 of 9	15 of 24	25 of 40
Disease duration: onset first symptoms to first balance test of study (years)	9.8 ± 8.2 (1–24)	10.2 ± 4.5 (4–21)	Not applicable
Weight (Kg) Height (cm) Body mass index	76.3 ± 12.9 (66–100) 166.5 ± 8.2 (160–176) 27.7 ± 5.6^#^ (22–38)	76.4 ± 17.5 (53–112) 171.8 ± 10.5 (160–187) 25.8 ± 5.3^#^ (19–37)	68.4 ± 7.5 (53–82) 173.9 ± 7.9 (160–180) 22.6 ± 1.5 (20–26)
**DISEASE MODIFYING TREATMENT**			
Natalizumab		10^*^	
Fingolimod	2	5	
No DMT	5	4	
Interferon beta-1b		2	
Glatiramer acetate	1	2	
Ocrelizumab		1	
Interferon beta-1a	1		

DMT, disease modifying treatment. Significant difference to healthy controls

# ,or between patient groups

* *p < 0.05. Controls weighed less and were slightly taller than the patients (see Table) resulting in a significant difference (p = 0.01) in body mass index. Unless otherwise mentioned there is no significant difference (2-sided t-test) between listed values of the subject groups*.

The patients' EDSS scores were assessed by a neurologist at relapse onset and also 3 months after their relapse. The remitting patients' EDSS scores were assessed by a neurologist within 2 weeks before or after every balance test.

Exclusion criteria for all patients were the inability to walk without a walking aid and the presence of orthopedic problems or other diseases/disabilities that could affect balance. Signed informed consent was obtained from all patients prior to the experiments. This study was approved (2014-026) by the Ethics Committee North-Central Switzerland (responsible for the University of Basel Hospital).

Balance of the patients was assessed by measuring trunk sway during a restricted sequence of six stance and gait tasks so as not to tire patients. All tasks were performed in the same order by each patient and executed without shoes. The tasks used were chosen based on previous studies in our laboratory comparing balance for 14 stance and gait balance tasks between MS patients and healthy controls (11, 20). Only those tasks with the strongest ability to discriminate patients from healthy control subjects were used. Trunk sway was measured with the SwayStar™ device (Balance International Innovations GmbH, Switzerland) that uses two gyroscopes to measure pitch (anterior-posterior) and roll (lateral) angular velocities of the lower trunk at a sample rate of 100 Hz. Angles were determined on-line by trapezoid integration of velocity signals. The device is worn at the level of L3-L5 in the middle of the lower back of the patients near the body's center of mass (21). The SwayStar™ device has been validated by a number of studies on MS patients (11, 20, 22).

Two one-legged stance tasks were performed for 20 s or until the patient lost balance. The patients were asked to use their better leg to stand on. The tasks were performed while standing on firm (S1EO) or foam (S1EOF) surface with eyes open. The S1EOF task was performed on foam to reduce the contribution of lower-leg proprioceptive to balance control. Afterwards the patients performed four walking tasks: a tandem gait task which was performed by walking 8 tandem steps with eyes closed (W8tanEC); walking on heels for 3 meters (W3mheels); walking 8 meters eyes open (W8mEO) and walking 8 meters eyes closed (W8mEC). Tasks were performed with eyes closed to eliminate visual inputs to balance control. At the beginning of each task the patients were asked to stand comfortably with feet hip-width apart to standardize the start of each test.

### Data processing and statistical analysis

To verify age matching of the remitting and control subjects a *t*-test was performed. No statistical test was performed for gender matching as the proportion of women was exactly equal for both groups (see Table 2). The outcome measures of balance assessments were peak-to-peak roll angle range (RAR), pitch angle range (PAR), roll angular velocity range (RVR), pitch velocity range (PVR) for the complete trial, and the task duration. To investigate whether the EDSS scores were correlated with trunk sway measures, a 1-step linear regression analysis was performed. To compare the patients during remission with the patients having a relapse a 2 × 2 ANOVA was performed on data at relapse onset, 0 months for remitting patients and at 3 months for both groups followed by *post-hoc t*-tests after verifying the assumption of normality for the data with a Kolmogorov–Smirnov test. Longitudinal balance changes in the remitting patients were analyzed using a mixed model analysis with a Bonferroni correction. Balance changes during and after a relapse in relapse patients were determined with the Wilcoxon signed-ranks test because of the smaller sample of relapse than remitting patients. Comparison with healthy age-matched normal values was performed using an independent-samples *t*-test for the remitting patients and the Mann–Whitney *U*-test for the relapsing patients again because of the small sample size. SPSS software was used for statistical analysis.

## Results

### Effect of relapses on balance in comparison to remitting-phase RRMS patients

**Figure 1:**
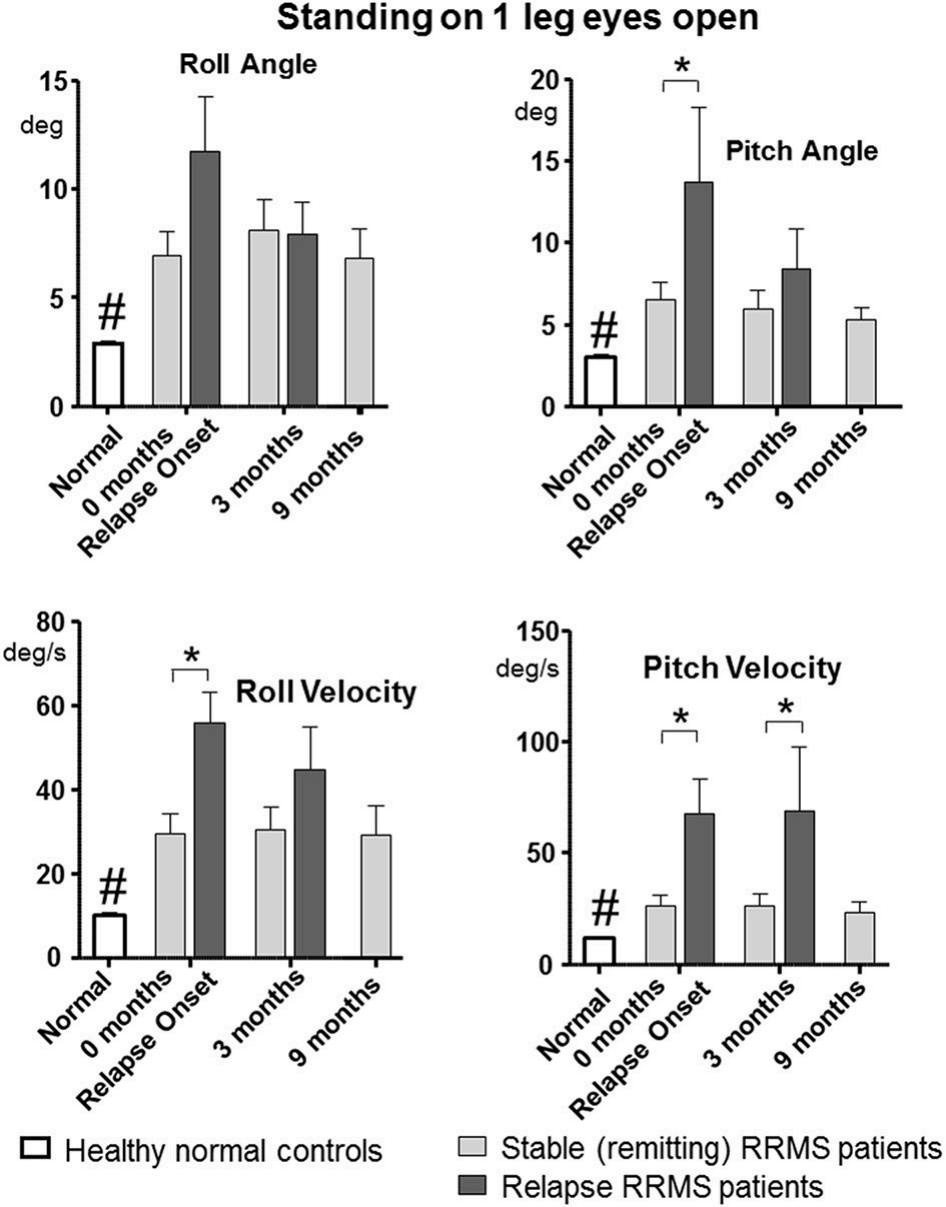
Peak-to-peak balance measures of the one-legged stance task on firm surface for remitting and relapse RRMS patients and normal age-matched controls. Data represented as mean with standard error of mean (sem), *p < 0.05 between patient groups, ^#^*p* < 0.05 between healthy controls and remitting and relapse RRMS patients. *n* = 24 for remitting RRMS patients, *n* = 9 for relapse RRMS patients during relapse, *n* = 40 for healthy controls.

**Figure 2:**
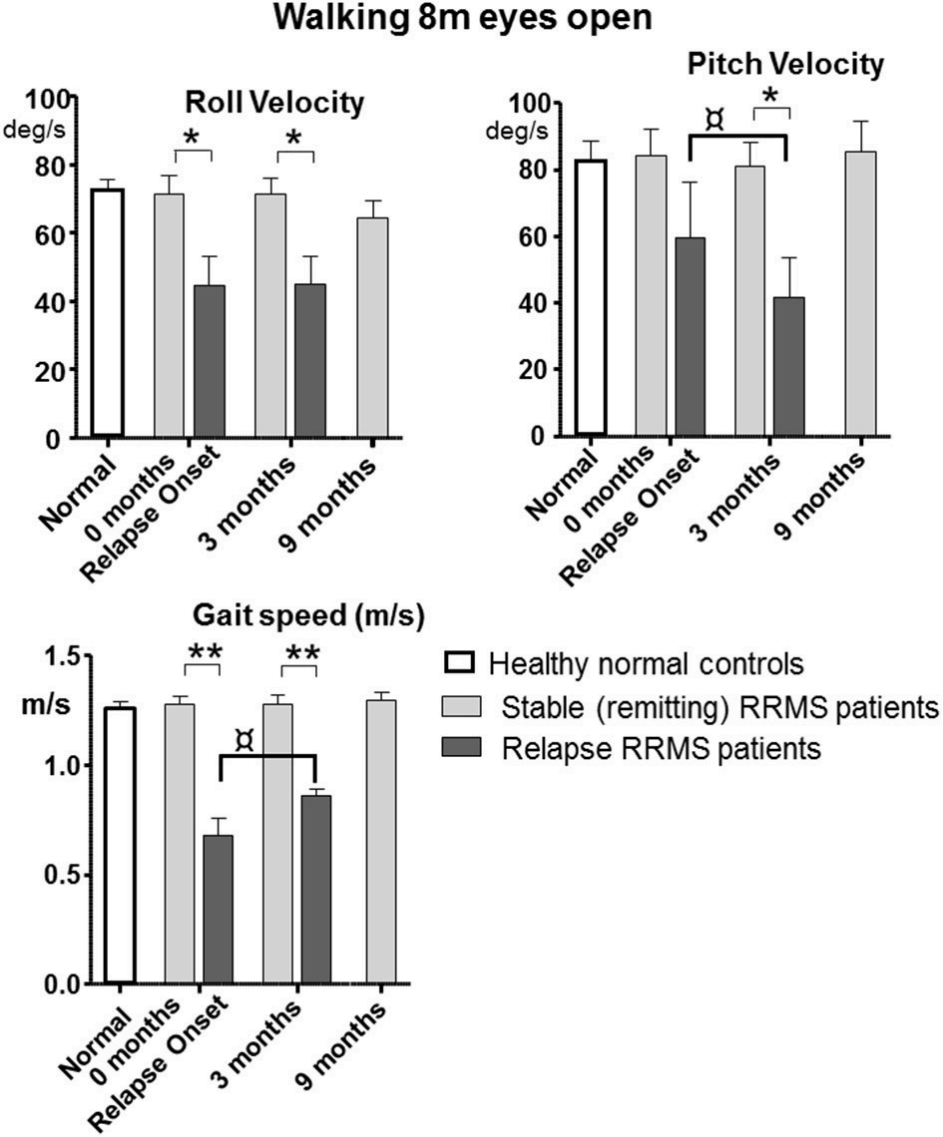
Peak-to-peak balance measures of walking 8 meters with eyes open for remitting and relapse RRMS patients as well as normal age-matched controls. Data represented as mean with sem, *p < 0.05, **p ≤ 0.001 between patient groups. ^¤^*p* < 0.05 between onset and 3 month values for relapse patients. *n* = 24 for remitting RRMS patients, *n* = 9 for relapse RRMS patients during relapse. Normal data *n* = 40 taken from Goutier et al. (18).

All tasks showed significant differences between the relapsing- and remitting-phase RRMS patients, except for one-legged stance on foam (Table 3). Figure 1 shows the deteriorated balance during the relapse phase with respect to remitting-phase patients for the one-legged stance task on a firm surface (*F* ≥ 2.6, 30 DOF, *p* < 0.05). All gait tasks showed increased duration (reduced gait speed) during a relapse (Table 3, *F* ≥ 11; 31 DOF *p* < 0.001). Relapsing RRMS patients showed not only decreased gait speed while walking 8 meters with eyes open (Figure 2) but also a reduction in pitch and roll velocities compared to the remitting-phase patients (see Figures 2). EDSS scores showed no significant differences (*p* > 0.05) between both patient groups at relapse onset and also 3 months later.

**Table 3:** Mean differences of balance measures between RRMS patients during remission (stable) (*N* = 24) and during relapse (*N* = 9) at relapse onset and 3 months after onset of a relapse.

**Stance tasks**	**Time of test**	**Balance measures**				
		**Duration (s)**	**RAR**	**RVR**	**PAR**	**PVR**
S1EO Diff	Onset	+2.59	+4.76^*^	+26.35^*^	+7.22^*^	+41.24^*^
Relapse to stable	3 months	+1.68	−0.23	+14.38	+2.45	+42.58^*^
S1EO stable Mean (sd)	0 months 3 months	14.46 (7.29)^#^ 14.31 (6.59)^#^	6.96 (5.37)^#^ 8.11 (6.82)^#^	29.39 (23.13)^#^ 30.55 (25.13)^#^	6.51 (5.48)^#^ 5.94 (5.71)^#^	26.39 (22.38)^#^ 26.50 (25.71)^#^
S1EO relapse Mean (sd)	0 months 3 months	17.05 (5.88) 16.00 (7.09)	11.73 (7.20)^¤^ 7.89 (4.05)^¤^	55.75 (20.75)^¤^ 44.93 (26.42)^¤^	13.73 (13.0)^¤^ 8.39 (6.61)	67.64 (44.66)^¤^ 69.08 (76.06)^¤^
Controls		19.82 (3.26)	2.84 (2.19)	8.99 (7.29)	2.89 (1.76)	10.34 (6.89)
S1EOF Diff	Onset	+4.85	+1.41	+9.36	+0.39	+33.56
Relapse to stable	3 months	+3.35	−2.91	−8.8	−0.41	−2.30
S1EOF stable Mean (sd)	0 months 3 months	11.10 (7.83)^#^ 12.71 (7.33)^#^	11.51 (8.47)^#^ 14.41 (9.93)^#^	47.87 (30.21)^#^ 50.06 (32.83)^#^	7.45 (4.77) 8.74 (6.17)	30.12 (16.62)^#^ 45.46 (51.30)^#^
S1EOF Rl Mean (sd)	0 months 3 months	15.96 (6.25) 16.06 (7.05)	12.91 (9.13)^¤^ 11.50 (9.73)^¤^	57.23 (34.31)^¤^ 43.25 (34.62)^¤^	7.84 (3.41) 8.25 (7.69)	63.68 (44.19)^¤^ 43.16 (40.50)^¤^
Controls		19.22 (3.30)	4.02 (4.38)	13.30 (13.36)	3.73 (3.65)	14.65 (12.61)
**Gait Tasks**		**Duration (s)**	**RAR**	**RVR**	**PAR**	**PVR**
W3mHls Diff	Onset	+8.12^**^	+3.54	−9.32	−0.89	−17.47
Relapse to stable	3 months	+1.88	+2.81	−15.83	−0.72	−12.00
W3mHeels St Mean (sd)	0 months 3 months	6.89 (2.34) 6.13 (2.22)	8.16 (2.85) 6.93 (1.95)	70.73 (17.45)^#^ 72.23 (17.97)^#^	12.35 (5.94) 9.05 (1.89)	88.30 (38.68)^#^ 86.19 (35.35)^#^
W3mHeels Rl Mean (sd)	0 months 3 months	15.01 (9.9)^¤^ 8.01 (4.45)	11.70 (11.86)^¤^ 9.75 (6.25)	61.41 (18.43) 56.4 19.82)	11.46 (6.43) 8.32 (2.05)	73.32 (32.37) 75.74 (18.59)
Controls		6.77 (1.78)	5.50 (1.65)	53.17 (9.40)	7.77 (1.95)	60.73 (14.45)
W8mEO Diff	Onset	+7.55^**^	+1.17	−27.04^*^	−1.02	−24.59
Relapse to stable	3 months	+2.88^**^	+2.99	−26.49^*^	+0.08	−39.37^*^
W8mEO St Mean (sd)	0 months 3 months	6.41 (1.06) 6.45 (1.20)	5.67 (2.28) 5.86 (1.81)	71.46 (26.64) 71.39 (17.97)	7.52 (2.22) 7.10 (1.89)	84.11 (39.28) 80.98 (35.19)
W8mEO Rl Mean (sd)	0 months 3 months	13.96 (7.11)^¤^ 9.34 (0.83)^¤^	6.83 (2.74) 8.85 (4.31)	44.42 (26.17)^¤^ 44.90 (22.31)^¤^	6.50 (3.21)^¤^ 7.09 (3.93)	59.93 (50.55) 41.61 (31.31)^¤^
Controls		7.25 (1.02)	6.34 (1.21)	70.28 (24.13)	9.84 (1.54)	77.24 (19.89)
W8mEC Diff	Onset	+11.33^**^	+1.23	−13.26^*^	+0.56	−24.0^*^
Relapse to stable	3 months	+0.77	+3.69	+5.52	+3.6	−7.41
W8mEC	0 months	12.00 (5.06)	6.28 (2.04)	57.76 (23.74)	8.20 (3.01)	72.84 (33.40)
St Mean (sd)	3 months	12.09 (4.62)	5.89 (2.01)	55.39 (17.35)	7.35 (1.80)	65.50 (27.35)
W8mEC	0 months	23.33 (14.16)^¤^	7.52 (3.97	44.52 (5.49)	8.26 (3.86)	48.95 (23.7)
Rl Mean (sd)	3 months	12.85 (3.72)	9.58 (6.72)	60.91 (17.72)	10.95 (6.26)	58.09 (20.06)
Controls		12.06 (1.04)	5.58 (1.59)	42.79 (15.66)	7.65 (2.46)	49.86 (18.13)

*Means and standard deviation (sd) of balance measures of remitting RRMS patients are listed at 0 and 3 months later below the differences. Values of healthy controls (N = 40) are listed below those of the remitting RRMS patients*.

*Positive difference values: value of relapsing RRMS patients greater than remitting RRMS patients. Negative difference values: value of relapsing RRMS patients less than remitting RRMS patients*.

Level of significance:

* *p < 0.05*,

** *p ≤ 0.001 between patient groups*,

# p < 0.05 between controls and remitting RRMS patients,

¤ *p < 0.05 between controls and relapse RRMS patients*.

*RAR, roll angle range; RVR, roll velocity range; PAR, pitch angle range; PVR, pitch velocity range; S1EO, one-legged stance with eyes open; S1EOF, one-legged stance with eyes open on foam surface; W3mHls, walking 3 m on heels; W8mEO, walking 8 meters with eyes open; W8mEC, walking 8 meters with eyes closed, remitting phase RRMS patients, Relapse or Rlapse or Rl Relapse phase RRMS patients*.

### Changes in balance control over time for remitting and relapse patients

Patients during remission showed no differences in trunk sway (*p* > 0.05) over the 9 months observation period (4 measurements) in any balance tasks (see Figures 1, 2 for examples of mean values for a stance and a gait task). Stance tests for remitting RRMS patients indicated worse balance control with respect to healthy controls (*p* < 0.05). In contrast, gait balance control did not differ from that of healthy controls (*p* > 0.05), except for walking on the heels (Table 3; Figures 1, 2). Significant changes in balance control over time were observed for relapse patients. Improvement (with respect to the values of remitting-phase patients) for the standing on one leg eyes open on a firm surface task took < 3 months for all balance measures except for pitch velocity (see Figure 1). At onset, relapse patients mostly showed decreased gait speed for all gait tasks compared to remitting-phase patients but increased gait speed (*p* < 0.05) to levels of remitting patients 3 months after the relapse (see Table 3). However, for walking eyes open, pitch velocity decreased even further at 3 months. Roll velocity and gait speed then remained less than that of remitting patients (*p* = 0.018, Figure 2; Table 3). Walking 3 meters on heels showed a significant increase in gait speed between onset and 3 months after a relapse (*p* = 0.028). Walking 8 meters with eyes closed showed a significant increase in gait speed over 3 months with a simultaneous increase in pitch and roll velocity (*p* ≤ 0.02) between onset and 3 months. EDSS scores of both relapsing and remitting patients showed no differences over time. Thus, although there was a general improvement toward the amplitudes of remitting-phase RRMS patient balance measures, several stance and gait measures of relapse patients required more than 3 months to improve to remitting patient levels.

The results of walking 8 tandem steps eyes closed were not analyzed due to task difficulty: 25% of remitting-phase patients fell or said they could not perform the task during the first visit and 30% in the second visit. Sixty-seven percent of the relapsing patients lost balance when tested at relapse onset and 43% 3 months later while performing this task. Although the number of patients is limited, the lower 24% lower rate of relapse patients who were at risk to fall after 3 months is also a clinical expression of the balance improvement after the relapse.

### Regressions of EDSS scores with balance measures over time in RRMS patients during remission and during relapse

A possible relationship between EDSS scores and trunk sway amplitudes in remitting-phase and relapse patients was studied with regression analysis (see Table 4). Generally, regression coefficients, *R*, were approximately 0.4 (*R*^2^ = 0.16) with some exceptions (see Table 4). EDSS scores of remitting-phase patients were correlated with two or more balance measures for all tasks except walking with eyes open (see Table 4). EDSS scores of relapse patients were only correlated with roll velocity for the task walking with eyes closed (W8mEC).

**Table 4:** Regression analysis of EDSS scores of remitting and relapsing RRMS patients with balance measures.

**Task**	**Score**	**RAR**	**RVR**	**PAR**	**PVR**
S1EO	EDSS Remitting	0.37^*^	0.45^**^	0.37^*^	0.43^**^
	EDSS Relapsing	ns	ns	ns	ns
S1EOF	EDSS Remitting	0.33^*^	0.39^*^	ns	ns
	EDSS Relapsing	ns	ns	ns	ns
W3mHeels	EDSS Remitting	0.37^*^	0.28^*^	0.39^*^	ns
	EDSS Relapsing	ns	ns	ns	ns
W8mEO	EDSS Remitting	ns	ns	ns	ns
	EDSS Relapsing	ns	ns	ns	ns
W8mEC	EDSS Remitting	0.37^*^	0.36^*^	0.31^*^	0.30^*^
	EDSS Relapsing	ns	0.61^*^	ns	ns

Regression coefficients, R, are presented. Significance

* *p < 0.05*,

** *p ≤ 0.001, ns not significant. N = 24 for remitting RRMS patients, n = 9 for relapsing RRMS patients*.

*RAR, roll angle range; RVR, roll velocity range; PAR, pitch angle range; PVR, pitch velocity range; S1EO, one-legged stance with eyes open for 20 s; W8tanEC, walking 8 tandem steps with eyes closed; S1EOF, one-legged stance with eyes open on foam surface for 20 s; W3mHeels, walking 3 meters on heels; W8mEO, walking 8 meters with eyes open; W8mEC, walking 8 meters with eyes closed; EDSS, Expanded Disability Status Scale*.

Data for cognitive status and fatigue assessed by using the functional systems (FS) score for cerebral function as part of the EDSS were available for 19 of the 24 remitting patients. The median FS score for cerebral function was 2 (±0.92), corresponding to mild decrease in mentation or moderate or severe fatigue.

## Discussion

The primary objective of this pilot study was to compare balance control recorded during stance and gait tasks for RRMS patients during remission with the balance of RRMS patients at relapse onset and 3 months later. Based on our previous research with vestibular neuritis patients (14, 15) we expected that 3 months would be sufficient for central compensation processes to improve balance for RRMS patients after a relapse. The longer than 3 months' time period required by the relapse patients suggests that future studies will need longer follow-up periods to track improvements in balance after a MS relapse. Additionally, in this study, remitting-phase MS patients were followed over 9 months. We could observe that balance control and EDSS scores of these patients indeed did not change and their balance scores could serve as a basis for the recovery level to be acquired by the relapse patients. The second objective of this study was to determine whether balance measures in remitting and relapse patients are related to their EDSS scores. We found significant regressions for remitting patients as in our previous study (11) but a lack of significant regressions for relapse patients with the exception of roll velocity for the task of walking eyes closed.

The results in relapsing patients showed improved gait speed 3 months after relapse onset for all gait trials. The changes (see Table 3) such as decreases in pitch velocity and roll velocity at 3 months could be described as relatively improved balance control because gait speed increased as well. This interpretation is based on the observation that increasing gait speed usually causes increased trunk sway velocity (18). It should be noted, however, that the improvement at 3 months was still not to the level of remitting RRMS patients who had trunk sway velocities equal to those of healthy controls (see Figure 2). The lower levels of sway amplitude and gait speed in relapse patients at 3 months were very similar to those of non-inflammatory polyneuropathy patients (16). Moreover, the time course of recovery is different for MS relapse patients from that of vestibular neuritis patients. The latter have greater than not lower than normal trunk sway velocity amplitudes during gait tasks at acute onset (14, 15), suggesting that a different type of central recovery process is initiated with MS relapse onset.

Despite the changes in balance measures, EDSS scores of the relapsing patients showed no correlation with balance measures for the two measurements of the relapse-phase. The remitting-phase patients showed significant correlations between balance measures and EDSS scores. Therefore, these results indicate that for judging the specific balance changes underlying a relapse, balance measures of remitting patients are best used as a comparison, as illustrated in Figures 1, 2, rather than EDSS scores. We assume that the reason for the absence of balance changes over 9 months in the remitting-phase patients is because MS is a lifelong disease where disability is accumulated over time. Thus, it is possible that our observation period of 9 months was too short to catch subtle changes in balance during the remitting-phases of RRMS.

In contrast to the present results with remitting-phase RRMS patients, a variation in between-visit balance parameters has been observed in a cohort consisting not only of patients with RRMS but also patients with progressive forms of MS (23). A variation in but not definitive progression of disability seems to be a key feature of a period of diagnostic uncertainty regarding the transition from relapsing remitting MS to secondary progressive MS (24). As the mean duration of this period of diagnostic uncertainty is 2.9 years (24) and in view to future different treatment strategies for patients with relapsing remitting and progressive forms of MS, it would be very useful to have paraclinical markers like posturography to define the typical duration of a relapse and use this duration to ascertain whether a possible transition from relapsing remitting to progressive MS occurred. Here we have attempted to define a typical pattern of balance variability with respect to remitting-phase RRMS patients in relapsing patients. For this pilot study our tracking interval of 3 months turned out to be too short to define exactly the typical improvement time-course of a relapse to remitting RRMS levels. Nonetheless, we have established that a tracking period of at least 3–4 months is required. Patient improvement has been investigated for acute vestibular loss subjects and shows stance and gait recovering to healthy normal levels with an exponential time course within 6–10 weeks (<3 months) after acute onset (15). Failure to recover within 12 weeks would then be assumed to be due to a lack of central compensation in these patients. In the case of MS patients we can hypothesize that not returning to levels of remitting patients for a remission lasting over 6 months could be indicative of a transition to progressive MS.

Comparison of patients during relapse with patients during remission showed an increase in sway with all balance measures for the one-legged stance task on firm surface during the relapse. In contrast, the decreased sway velocities during gait tasks would seem to indicate that the compensation during a relapse is more effective for gait than stance (compare Figures 2A,B). This finding, however, is associated with reduced gait speed (Figure 2C) indicating that patients compensated for their decreased balance abilities by decreasing gait speed (18), and emphasizes that both gait speed and trunk motion need to be measured in order to estimate improvements in balance control for gait.

EDSS scores showed no difference between remitting-phase patients and relapsing patients. Moreover, 3 months after relapse onset, balance of relapsing patients improved, while EDSS scores remained unchanged. Martin et al. also showed that patients can experience balance impairments while their EDSS score is minimally altered (12), as observed in this study. Thus, we would propose that it becomes more and more important to measure not only the worsening of symptoms in MS patients but also their improvement as emerging clinical MS trials test neuroprotective substances to restore function after relapses (25). Because the EDSS is the only clinical outcome measure for disability accepted by health authorities for approval of MS therapeutics and this did not change over 3 months from relapse onset, it would seem important to supplement the standard clinical examinations by testing the patients' balance and gait capabilities in order to document an improvement after a relapse.

Corporaal et al. have shown that EDSS scores correlate highly (*R* = 0.7) with balance measures of trunk sway (11). The results of this study documented significant regressions for EDSS scores too, with balance measures accounting for 28 to 45% of the variance in EDSS scores. However, the highest R found with the balance measures was 0.45 probably indicating a statistical relationship and a weaker clinical relationship between the balance measures of trunk sway and EDSS scores. Comparing these current results with those of Corporaal et al. who had a larger range of EDSS scores (up to 4.5) implies that balance measures of trunk sway can have a more significant relationship with EDSS scores if the EDSS score range is large, however not for the small range of EDSS scores in this study. Longitudinal regression of EDSS with balance measures of trunk sway in relapsing patients showed a significant regression (*R* = 0.61) only for roll velocity in the walking 8 meters with eyes closed task. This may indicate that higher correlations can be expected with sensory deprivation (eyes closed). All other balance tasks showed no significant correlation between balance measures and EDSS values of relapse patients. Thus, we cannot exclude a by chance relationship for this eyes closed walking test.

The range of balance tasks performed in this study was limited. In limiting the number of tasks we were aware that MS patients have a limited stamina and fatigue which is an important confounding factor in balance measurements (1). Similar to previous studies that showed an association between impaired cognition/fatigue and impaired balance, based on data obtained from a part of the cohort, our remitting patients also showed signs of decreased mentation or moderate or severe fatigue as measured by the appropriate FS score of the EDSS (26–29). However, in this respect it is important to note that patients in this study did not state that the tasks were fatiguing. Nonetheless, this is an aspect that needs to be investigated in future studies. One possible limitation of our study was the lower proportion of women in the remitting compared to the relapse group of patients. In our previous studies on balance control we have found no gender differences (1) for the tests we used. An exception occurs when young people are asked to walk at a faster than normal speed (1). However, there are no gender difference when young subjects walked, as in our current tests, at their preferred speed (1). Another limitation of our study was the small number (9) of relapse patients studied and that the relapse symptoms were not uniform. Due to the small number of relapse patients it is not possible to assign our findings to brain or spinal cord lesions. Future studies should therefore expand the number of patients studied rather than the number of balance tasks.

This pilot study was designed under the assumption that central compensation processes require approximately 3 months to compensate for a balance deficit (14, 15). We observed a worsening in balance control at relapse onset which recovers toward the remitting phase levels at approximately 3 months. Therefore, it would be interesting to follow relapsing patients over a slightly longer time, possibly 4 months with shorter intervals of 1 month between test dates in order to plot the dynamics of their improvement to the levels of remitting-phase patients more accurately. The question arises if the recovery will reach the level of remitting-phase patients (the presumed pre-relapse level) after 4 months or not. As relapses normally cause accumulating CNS damage (7), it might be expected that relapsing patients will never reach pre-relapse levels, equivalent to levels of remitting-phase RRMS patients, for all balance measures. As mentioned above, future work should determine in detail when improvement of relapsing patients reaches the levels of the remitting-phase patients.

Regarding the practical consequences of our study for estimating the dynamic characteristics of relapses, we have demonstrated that posturography measures obtained with body mounted sensors are sensitive enough to detect subtle changes in neurological status which were not detected with EDSS scores, the most widely used clinical instrument to monitor disease progression in MS patients. Other authors have come to similar conclusions. For example Solomon et al. (30) showed that wireless, body-mounted sensors could detect postural sway abnormalities in minimally disabled MS patients prior to their developing a clinically evident disability or impaired gait speed. Furthermore, such wireless wearable devices provide excellent reliability (31), and are not as clumbersome and expensive as non-wearable systems (32). Thus, translating these objective markers of disease severity obtained from wearable inertial devices into routine clinical practice would seem to be of high relevance in planning MS treatment. As balance impairment is one of the most prominent and feared symptoms of MS it is recommended that a balance measurement with wearable devices be performed routinely in the clinic in addition to EDSS scores. However, further research is needed to investigate which balance tasks would be the most optimal for clinical use in identifying the balance deficits of relapse patients with respect to remitting-phase RRMS patients.

## Data availability statement

The raw data supporting the conclusions of this manuscript will be made available by the authors, without undue reservation, to any qualified researcher.

## Author contributions

OF, ÖY, and JA contributed to the research concept and the acquisition, analysis, and interpretation of the data. They drafted and revised the work critically. They agree to be accountable for all aspects of the work in ensuring that questions related to the accuracy or integrity of any part of the work are appropriately investigated and resolved. DT, HR, and AS contributed to the acquisition and analysis of data for the work. OF, DT, HR, AS, ÖY, and JA provide approval for publication of the content.

### Conflict of interest statement

The author JA declares a conflict of interest as he worked as a consultant for the company producing the SwayStar equipment used in this study. The remaining authors declare that the research was conducted in the absence of any commercial or financial relationships that could be construed as a potential conflict of interest.
